# Malignant Melanoma Arising in a Congenital Melanocytic Nevus in Early Infancy With Fatal Metastatic Progression: A Case Report

**DOI:** 10.1155/crom/1159345

**Published:** 2026-11-14

**Authors:** Rasoul Goli, Nasrin Karimi, Fereshteh Seyed Mohammad Shahi, Ali Soheyli

**Affiliations:** ^1^ Department of Medical-Surgical Nursing School of Nursing and Midwifery Urmia University of Medical Sciences Urmia Iran umsu.ac.ir; ^2^ Department of Nursing School of Nursing and Midwifery Hamadan University of Medical sciences Hamadan Iran umsha.ac.ir; ^3^ Department of Midwifery Ahvaz Jundishapur University of Medical Sciences Ahvaz Iran ajums.ac.ir; ^4^ Department of Nursing School of Nursing and Midwifery Golestan University of Medical Sciences Kalaleh Iran goums.ac.ir

**Keywords:** case report, congenital melanocytic nevus, histopathology, infancy, melanoma

## Abstract

Malignant melanoma arising within a congenital melanocytic nevus (CMN) during early infancy is rare and presents substantial diagnostic and therapeutic challenges. We report a male infant with a congenital pigmented lesion on the right cheek that developed progressive darkening, rapid enlargement, nodularity, ulceration, and intermittent bleeding during the first 3 months of life. Histopathological examination revealed melanoma associated with a CMN, with a Breslow thickness of 2.5 mm and ulceration. Distinguishing melanoma from a benign proliferative nodule was a central diagnostic consideration. Surgical excision was performed at 4 months of age, with microscopically negative margins. The facial location complicated surgical planning, and omission of sentinel lymph node biopsy left pathological nodal status undetermined. Although no recurrence or metastatic disease was detected during the initial 6 months of postoperative follow‐up, the child subsequently died. This case highlights the diagnostic complexity and potentially serious outcomes of melanoma arising in CMN during infancy. It emphasizes the importance of prompt assessment of changing congenital lesions, expert histopathological review, multidisciplinary management, and prolonged surveillance. An initially reassuring postoperative assessment should not be interpreted as evidence of durable disease control.

## 1. Introduction

Congenital melanocytic nevi (CMNs) are melanocytic lesions present at birth. Melanoma risk varies with the phenotype and extent of congenital involvement; a single risk estimate should not be applied to all CMN [1]. Melanoma can occur during childhood; however, its recognition in infancy is complicated by benign proliferative nodules that can resemble malignancy [2]. This report describes an infant with a changing facial CMN and a diagnosis of melanoma. The clinical course is presented with attention to diagnostic evidence, surgical constraints, incomplete nodal staging, and the limits of early postoperative surveillance.

## 2. Case Presentation

### 2.1. Initial Presentation and Nevus Characteristics

A male infant was delivered at term following an uneventful pregnancy. There was no known family history of malignancy, and no identifiable prenatal or perinatal risk factors were reported. At birth, a flat, well‐demarcated pigmented lesion measuring 4 × 5 cm was observed on the right cheek. The lesion was smooth, homogeneous in color, and nonulcerated, clinically consistent with a CMN (Figure 1).

**Figure 1:**
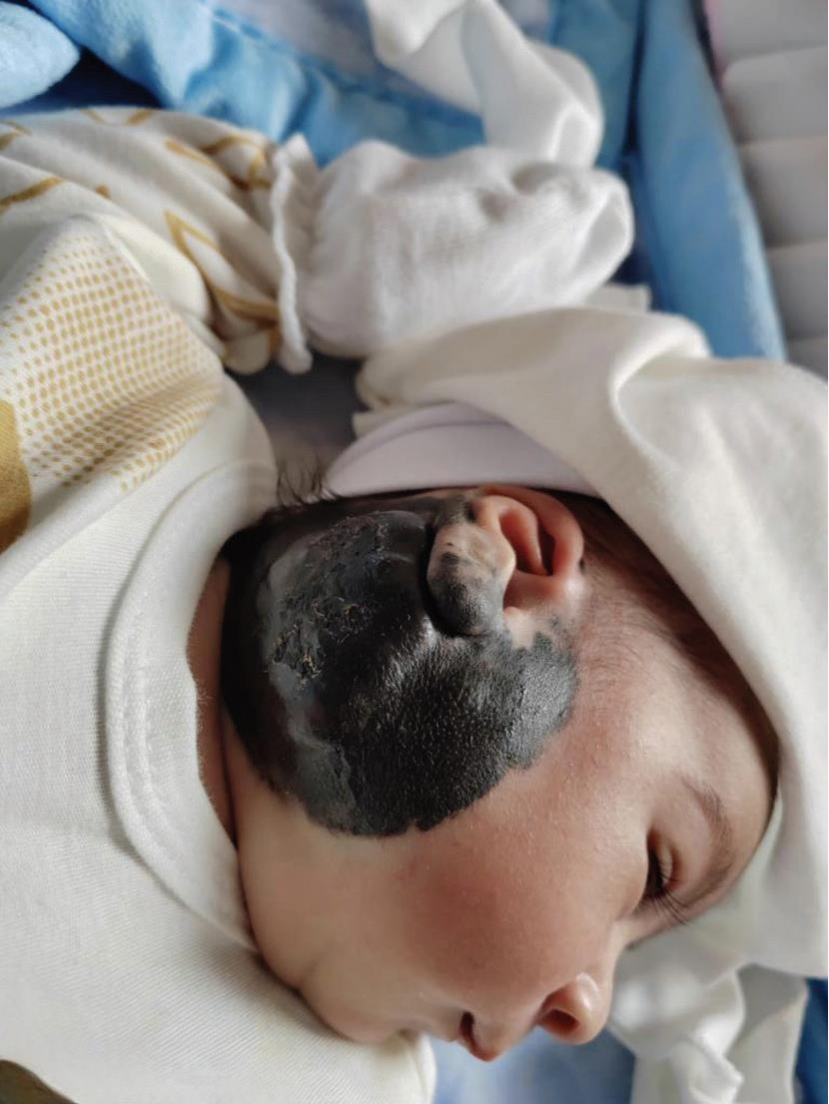
Congenital pigmented lesion at birth.

The estimated projected adult diameter was approximately 8.5 cm, corresponding to the medium M1 category of the Krengel classification. No satellite nevi, hypertrichosis, surface rugosity, or palpable dermal nodules were documented at birth.

### 2.2. Clinical Progression and Diagnostic Evaluation

At approximately 2 months of age, progressive darkening and slight thickening of the lesion became apparent. By 3 months, the lesion showed rapid enlargement, nodularity, surface ulceration, and intermittent bleeding. The parents reported growth disproportionate to the child′s overall somatic growth (Figure 2).

**Figure 2:**
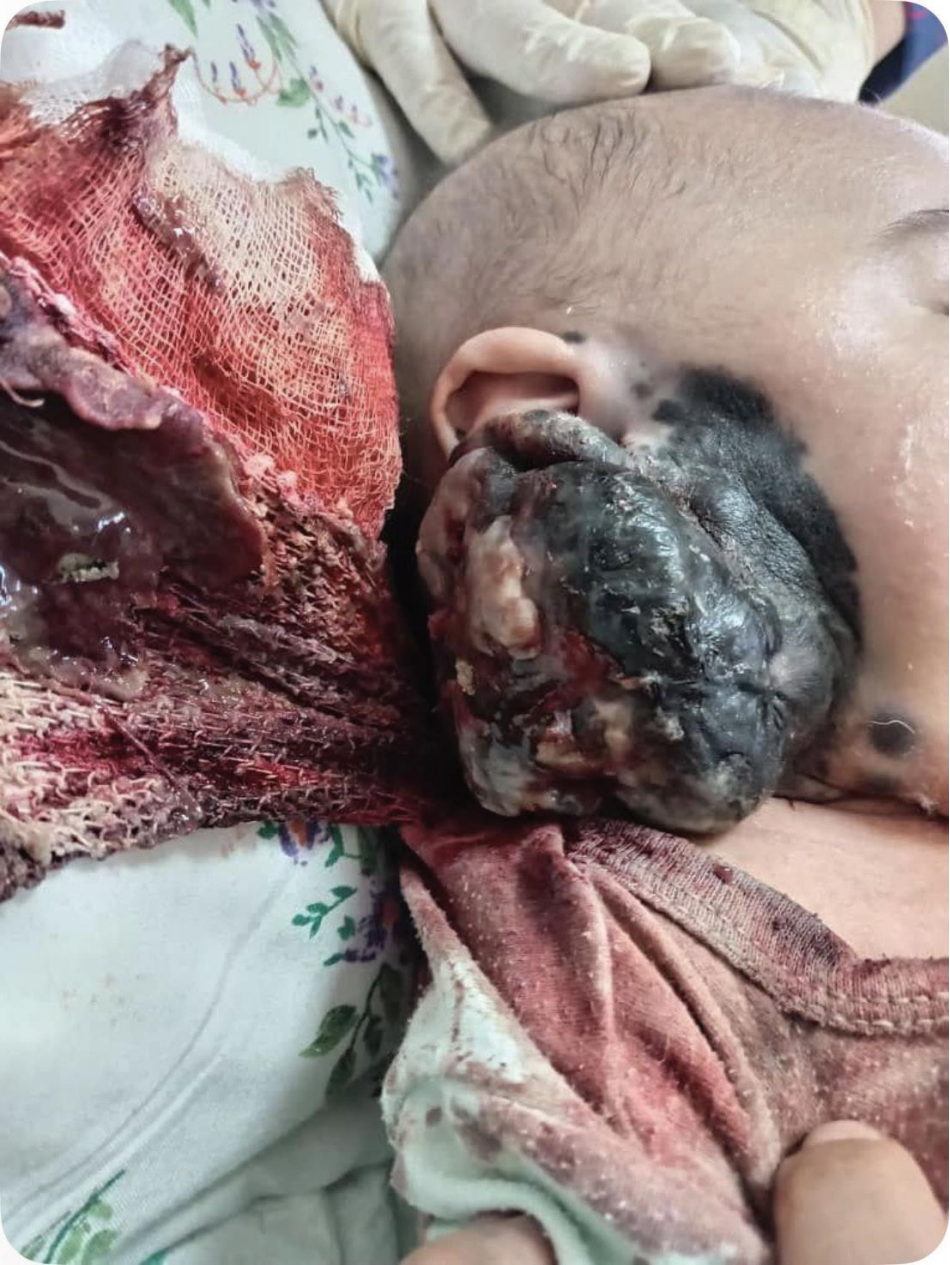
Clinical appearance after reported progression.

Urgent dermatological evaluation was undertaken. Dermoscopy demonstrated irregular pigmentation and atypical vascular patterns. At 3 months of age, an incisional biopsy of the most clinically concerning nodular area was performed to establish the diagnosis before definitive surgery.

### 2.3. Histopathological and Molecular Findings

Histopathological examination demonstrated an asymmetric melanocytic proliferation associated with a CMN. The atypical component comprised irregular nests and confluent dermal aggregates showing marked nuclear pleomorphism, prominent nucleoli, and loss of maturation with increasing depth. Breslow thickness was 2.5 mm, with ulceration and Clark Level IV invasion. The mitotic rate was 8 mitoses/mm^2^, including deep and atypical mitotic figures. No lymphovascular invasion was identified.

Immunohistochemical examination showed diffuse SOX10 positivity, persistent HMB‐45 expression in the deeper component, a Ki‐67 proliferation index of approximately 30%, and loss of p16 expression within the malignant component. The diagnosis was based on the combined architectural, cytological, and immunohistochemical findings, with particular consideration of the differential diagnosis of a benign proliferative nodule. Representative histopathological and immunohistochemical findings from the patient′s specimen are shown in Figure 3. The comparative immunohistochemical panels in Figure 3D show the malignant component and an associated benign proliferative nodule component within the congenital nevus; the latter showed lower proliferative activity and retained p16 expression.

**Figure 3:**
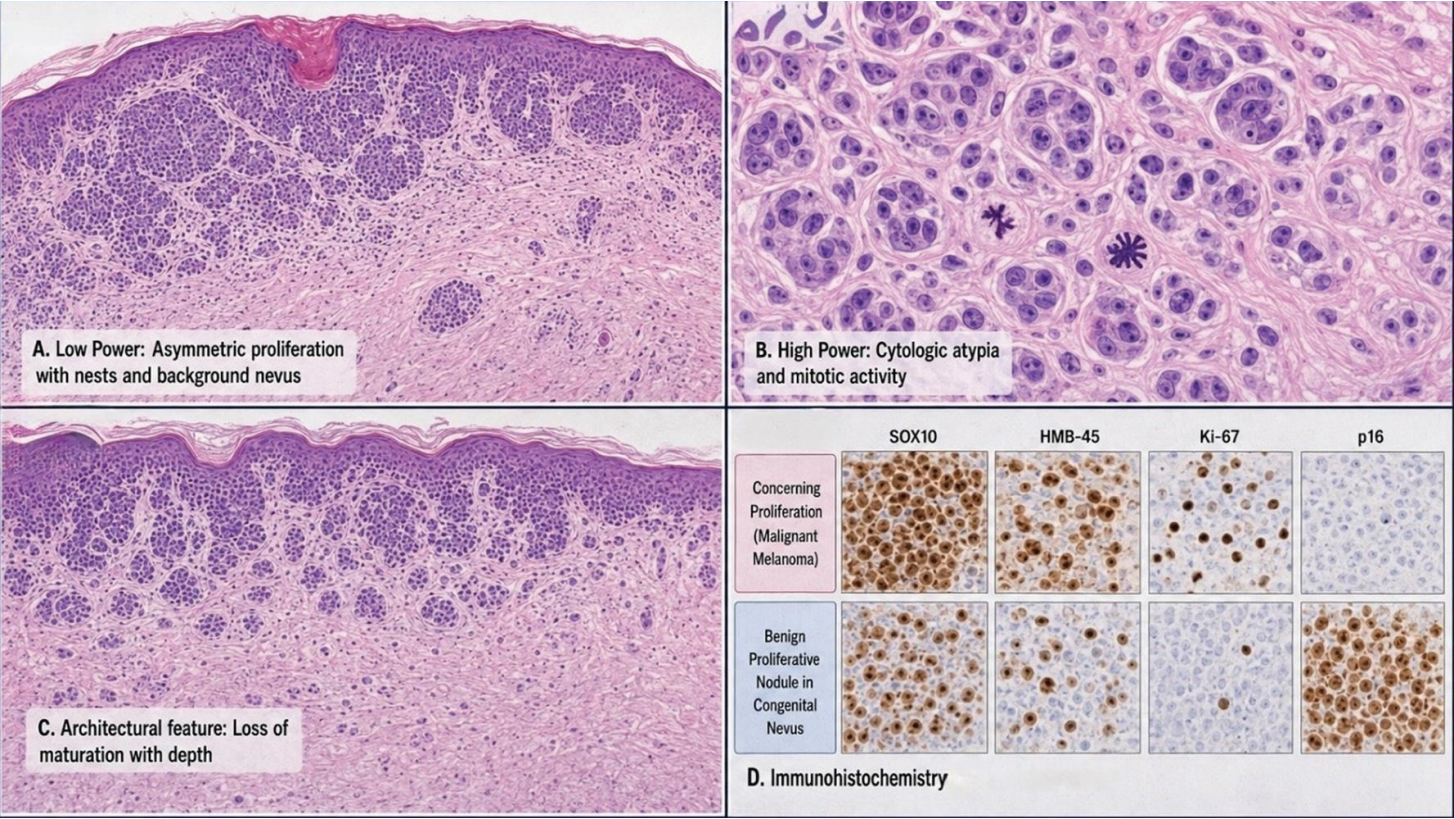
Histopathological and immunohistochemical findings in the patient′s specimen. The original digital composite did not retain objective magnifications or calibrated scale bars; none has been retrospectively assigned. (A) Low‐power H&E view demonstrating an asymmetric melanocytic proliferation composed of irregular nests adjacent to the background congenital nevus. (B) High‐power H&E view showing cytologic atypia, nuclear pleomorphism, prominent nucleoli, and increased mitotic activity. (C) Architectural assessment demonstrating loss of melanocytic maturation with increasing dermal depth, a feature concerning for malignancy. (D) Immunohistochemical findings in the patient′s specimen, comparing the malignant melanoma component with the benign proliferative nodule component.

Molecular and genetic testing was not performed because it was unavailable at the treating center; consequently, the tumor′s mutational status remained unknown.

### 2.4. Staging and Multidisciplinary Assessment

Regional ultrasonography showed no suspicious lymph nodes. Baseline abdominal ultrasonography and chest radiography showed no detectable metastatic disease, although these investigations could not exclude occult metastases.

The case was reviewed by pediatrics, pediatric oncology, dermatology, and plastic surgery. The team considered the pathological diagnosis, rapid clinical progression, facial location, and anticipated functional consequences of surgery. Definitive excision was selected to obtain local tumor clearance while preserving adjacent facial structures.

Sentinel lymph node biopsy was discussed but was not performed because of the team′s concerns about the technical challenges of facial lymphatic mapping and additional procedural morbidity in a very young infant. These were case‐specific considerations rather than a general contraindication based on age. Occult nodal involvement therefore remained possible.

The primary tumor was classified as pT3b according to the eighth edition of the AJCC staging system, based on its 2.5‐mm Breslow thickness and ulceration. Pathological nodal status remained undetermined, and no complete pathological stage group was assigned.

### 2.5. Surgical Management

At 4 months of age, the patient underwent wide local excision under general anesthesia. The plastic surgery team participated in preoperative planning, performed the excision and reconstruction, and supervised early postoperative surgical assessment. The lesion measured approximately 6.0 × 5.5 cm and involved the right cheek and preauricular region. No gross involvement of the external ear, oral commissure, underlying facial musculature, or parotid gland was observed.

A gross peripheral margin of approximately 1 cm was planned, with further narrowing in the preauricular region to preserve adjacent structures. The surgical team considered that a wider excision would increase the risk of distortion of the external ear and oral commissure and substantially enlarge the reconstructive defect. The selected margin therefore represented an individualized anatomical compromise. The specimen was removed en bloc, including the skin and subcutaneous tissue down to the superficial musculoaponeurotic system, and oriented with sutures marking the superior and anterior margins.

The resulting defect measured approximately 7.5 × 7.0 cm. Reconstruction was performed using a local cheek advancement flap with subcutaneous undermining and layered closure. This technique was selected to provide adjacent tissue coverage while minimizing tension and distortion of nearby facial landmarks. Particular attention was directed to protecting the facial nerve branches, parotid duct, external ear, and oral commissure. Deep tissues were approximated with absorbable sutures, and the skin was closed with fine nonabsorbable sutures. No drain was placed.

Estimated blood loss was 15 mL, and no intraoperative complications occurred. Definitive examination of the excision specimen confirmed residual melanoma associated with a CMN. Peripheral and deep margins were microscopically negative, with minimum clearances of 8 and 4 mm, respectively.

### 2.6. Postoperative Recovery and Surveillance

The postoperative course was uncomplicated. Wound care consisted of gentle saline cleansing and a nonadherent protective dressing. The parents received instructions on dressing care and signs requiring reassessment, including increasing redness, discharge, wound separation, and bleeding. The patient was discharged on the second postoperative day, and skin sutures were removed on postoperative Day 7.

At the 2‐week review, the flap was viable, and the wound had healed without infection, hematoma, or dehiscence. No clinically apparent facial weakness or impairment of oral competence was documented.

Follow‐up examinations were scheduled monthly during the first six postoperative months, with regional nodal ultrasonography every 3 months. Pediatric oncology elected to continue surveillance without initial adjuvant systemic therapy after reviewing the available staging results and the uncertainties surrounding treatment in early infancy.

### 2.7. Follow‐Up and Outcome

At the 6‐month postoperative assessment, the surgical site had healed satisfactorily. Clinical examination and the investigations performed at that visit showed no detectable recurrence or metastatic disease. Nevertheless, this assessment did not establish durable disease control.

In this case, approximately 9 months after surgery, the patient developed an enlarging ipsilateral cervical mass and persistent cough. Ultrasonography and subsequent biopsy confirmed nodal metastatic melanoma, whereas chest computed tomography showed pulmonary lesions interpreted as metastatic disease. The patient received pediatric oncology assessment and supportive care, followed by palliative care as the disease progressed. The child died approximately 12 months after the initial diagnosis.

Table 1 summarizes the chronological course of the case, from identification of the congenital lesion at birth through clinical progression, diagnostic evaluation, surgical treatment, and follow‐up. It provides an overview of the key clinical events and the reported outcome.

**Table 1:** Clinical timeline.

Approximate time	Clinical event
Birth	A flat, well‐demarcated, nonulcerated pigmented lesion measuring 4 × 5 cm was observed on the right cheek, clinically consistent with a congenital melanocytic nevus.
Birth to 2 months	Routine infant care was provided, but no specialist dermatological assessment occurred before the onset of concerning changes.
Approximately 2 months of age	Progressive darkening and thickening of the lesion were first noticed.
Approximately 3 months of age	Rapid disproportionate growth, nodularity, ulceration, and intermittent bleeding prompted urgent dermatological evaluation.
Initial dermatological assessment, approximately 3 months of age	The infant weighed 6.0 kg and was feeding adequately, with no fever, lethargy, or clinically apparent systemic illness. Dermoscopy showed irregular pigmentation and atypical vascular patterns.
Within 1 week of the urgent assessment	A diagnostic biopsy was performed, approximately 4–5 weeks after the initial pigmentation changes and within 1 week of the reported ulceration and bleeding.
Within 1 week after biopsy; approximately 3–3.5 months of age	Histopathological examination confirmed melanoma with a Breslow thickness of 2.5 mm and ulceration. The pathology reporting interval was approximately 1 week.
Approximately 3.5–4 months of age	Initial staging investigations and multidisciplinary treatment planning were undertaken. Regional ultrasonography showed no suspicious lymph nodes.
Approximately 4 months of age	Surgical excision and wound closure were performed.
Postoperative Day 2	The infant was discharged following an uncomplicated immediate postoperative course.
Postoperative Day 7	Skin sutures were removed following surgical assessment.
Approximately 2 weeks after surgery	Wound healing was assessed, with no infection or dehiscence identified.
Approximately 10 months of age; 6 months after surgery	Clinical examination and the imaging performed showed no detectable local recurrence or metastatic disease. The calculated age assumes surgery at 4 months.
Photographic documentation during follow‐up	Postoperative photographs were unavailable to the authors. Whether serial photographs had originally been taken could not be established from the information available.
Approximately 13 months of age; 9 months after surgery	Symptoms suggestive of recurrence developed, prompting further assessment for metastatic disease.
Approximately 13–15 months of age	Oncological and supportive care were provided following disease progression.
Approximately 15–15.5 months of age; about 12 months after diagnosis	The child died following the subsequently reported metastatic progression. The calculated age assumes diagnosis at approximately 3–3.5 months.

## 3. Discussion

The educational value of this case lies in the diagnostic and management challenges of a rapidly changing facial congenital lesion during early infancy. It should not be presented as the first reported infantile melanoma or as proof that medium‐sized CMN generally have an aggressive course. A single case cannot establish incidence, comparative treatment effectiveness, or prognosis.

The principal diagnostic challenge in this case was distinguishing malignant melanoma from a benign proliferative nodule arising within a CMN. Proliferative nodules may exhibit rapid growth, cellular atypia, and mitotic activity and can therefore mimic melanoma, particularly during infancy. In the present case, the combination of asymmetric architecture, marked cytological atypia, increased mitotic activity, loss of maturation with depth, supportive immunohistochemical findings, and the subsequent aggressive metastatic course favored malignant melanoma. This case emphasizes that rapidly changing nodules within CMNs require prompt biopsy and expert dermatopathological evaluation.

Melanoma arising in CMN has previously been described in childhood. In this setting, clinicopathological correlation and expert review are important because benign proliferative nodules can show overlapping morphological features [2]. Clinical enlargement alone does not establish malignant transformation, and subsequent progression does not replace adequate review of the original tissue.

### 3.1. Rationale for Treatment Selection

In this case, surgery was selected because the biopsy‐confirmed melanoma was considered technically resectable, and the initial investigations had not identified clinically apparent regional or distant disease. Rapid enlargement, recurrent bleeding, and ulceration provided additional reasons to obtain prompt local control. Excision also permitted assessment of the complete specimen and surgical margins. The facial location required careful consideration of tumor clearance, preservation of adjacent structures, and the functional consequences of reconstruction.

A general cutaneous melanoma guideline recommends a 2‐cm clinical margin for melanomas thicker than 2 mm [3]. This provides a reference point rather than an infant‐specific mandate. The approximately 1‐cm margin used here was narrower and reflected an individualized operative decision. The surgeon confirmed that a wider excision was judged likely to compromise adjacent facial structures and substantially increase reconstructive morbidity. The microscopically negative margins do not demonstrate equivalence between the selected margin and a wider recommended margin.

Local surgery and adjuvant systemic therapy are separate treatment decisions. The treating pediatric oncology team elected surveillance without initial systemic therapy after considering the available staging findings, treatment‐related toxicity, and the limited evidence specific to early infancy. Young age alone was not regarded as an absolute contraindication to systemic treatment.

### 3.2. Sentinel Lymph Node Biopsy Considerations

The ASCO/SSO guideline recommends SLNB for clinically node‐negative intermediate‐thickness melanoma (> 1.0–4.0 mm) [4]. Thus, the reported 2.5‐mm ulcerated primary would ordinarily prompt discussion of sentinel nodal staging. These recommendations are not specific to early infancy, and age alone should not be described as an absolute contraindication.

The multidisciplinary team attributed omission of SLNB to concerns about facial lymphatic mapping and procedural morbidity. Potential intraparotid drainage and proximity to facial nerve branches were anatomical concerns considered during planning; lymphatic mapping was not performed.

This decision left pathological nodal status undetermined. Negative ultrasonography could not exclude microscopic nodal metastases and was not equivalent to sentinel node staging. Consequently, the omission of SLNB should be acknowledged as a limitation of staging rather than presented as evidence that nodal assessment was unnecessary.

### 3.3. Postoperative Care and Follow‐Up Considerations

Postoperative care should be tailored to the closure technique and the surgeon′s assessment. Practical priorities include appropriate wound protection, aseptic dressing changes, and monitoring for infection, hematoma, wound separation, and compromised tissue viability. Parents should receive clear instructions regarding wound care and prompt reassessment for increasing redness, swelling, discharge, persistent bleeding, fever, or changes in skin color around the reconstruction. Facial movement, eyelid closure, oral competence, and feeding should also be assessed where relevant to the operative site.

Early surgical review should evaluate wound healing and determine the appropriate timing of suture removal. Subsequent visits should assess scar evolution and facial function, whereas dermatological and oncological surveillance should address the operative site, remaining pigmented lesions, and regional lymph nodes. Follow‐up intervals and imaging should be individualized rather than presented as a universally applicable infantile melanoma protocol. Serial clinical photographs, when available and obtained with appropriate consent, can support assessment of healing and cosmetic changes. Importantly, satisfactory wound healing and an initially negative surveillance assessment do not establish durable oncological control.

Management was particularly challenging because of the patient′s early age, the facial location of the tumor, and the limited evidence available for melanoma treatment during infancy. Although complete local excision was achieved, sentinel lymph node biopsy was not performed, leaving pathological nodal status undetermined. In addition, molecular characterization of the tumor was unavailable. These limitations restricted complete biological and pathological characterization of the disease. The subsequent metastatic progression despite an initially reassuring postoperative assessment highlights the importance of prolonged multidisciplinary surveillance involving pediatrics, oncology, dermatology, surgery, and pathology. Postoperative photographs were unavailable, limiting independent visual assessment of sequential wound healing, scar evolution, and the final cosmetic outcome. The description of postoperative recovery is therefore restricted to the available clinical documentation. Satisfactory early wound healing should not be interpreted as evidence of an optimal long‐term cosmetic result or durable oncological control.

Molecular characterization was not reported in the supplied case account. This limits biological characterization and the assessment of potentially actionable alterations. Molecular or copy‐number analysis can complement expert histopathological review in diagnostically difficult CMN‐associated proliferations [2]. No mutational status or molecular explanation for progression can be inferred from morphology or immunohistochemistry alone.

## 4. Conclusion

Malignant melanoma arising within a CMN during early infancy is exceptionally rare and diagnostically challenging. This case highlights the importance of careful clinicopathological correlation, expert histopathological assessment, appropriate pathological staging, multidisciplinary management, and prolonged surveillance. An initially reassuring postoperative assessment does not exclude subsequent aggressive metastatic progression.

## Funding

No funding was received for this manuscript.

## Disclosure

An earlier version of this manuscript was made publicly available as a preprint on ResearchGate in July 2026 (10.13140/RG.2.2.30447.60327) [5].

## Consent

Written patient consent was obtained from the patient′s parents and/or legal guardians prior to submission of the case report.

## Conflicts of Interest

The authors declare no conflicts of interest.

## Data Availability

The data that support the findings of this study are available on request from the corresponding author. The data are not publicly available due to privacy or ethical restrictions.
